# A bibliometric analysis from 2013 to 2023 reveals research hotspots and trends in the connection between sport and regenerative medicine

**DOI:** 10.1097/MD.0000000000038846

**Published:** 2024-07-05

**Authors:** Lv Xiongce, Ye Tao, Jing Zhu, Yan Jin, Lixia Wang

**Affiliations:** a Department of Physical Education, Beijing University of Posts and Telecommunications, Beijing, China; b Department of Psychology, Russian Sports University, Moscow, Russia; c Department of Propaganda, Hebei University of Science and Technology, Shijiazhuang, China; d College of Sports, Shijiazhuang University, Shijiazhuang, China.

**Keywords:** bibliometric analysis, Citespace, knowledge mapping, regenerative medicine, sport

## Abstract

The field of regenerative medicine for sports injuries has grown significantly in the 21st century. This study attempted to provide an overview of the current state of research and key findings regarding the relationship between sport and regenerative medicine in general, identifying trends and hotspots in research topics. We gathered the literature from the Web of Science (WOS) database covering the last 10 years (2013–2023) pertaining to regenerative medicine for sporter and applied Citespace to assess the knowledge mapping. The findings demonstrated that there were 572, with a faster increase after 2018. The country, institution, and author with the most publications are the USA, Harvard University, and Maffulli Nicola. In addition, the most co-cited reference is *J Acad Nutr Diet* (2016) (199). Adipose tissue, high tibial osteotomy, and bone marrow are the hot spots in this field in the next few years.

## 1. Introduction

Without accounting for the expense of follow-up rehabilitation, the estimated annual cost of acute injuries in collegiate sports in the USA is about $1.5 billion. In addition to this enormous financial burden, sports injuries may end an athlete’s career in certain cases if a proper diagnosis and adequate interventions are not made. The number of women engaging in contact-based and pivoting sports, the number of middle-aged people returning to sports, and the rise in age-related injuries will all contribute to increased costs and detrimental effects on quality of life. In the realms of sports and medicine, it is imperative to maximize repair, recuperation, and function following an injury for those that are unpredictable and unavoidable. In an effort to speed recovery and ease return to sport, the field of regenerative medicine for sports injuries has grown significantly in the 21st century. These approaches utilize the current understanding of stem cell therapy and platelet-rich plasma (PRP) as promising means of repairing injuries.^[1,2]^

Innovative techniques are employed in regenerative medicine to investigate and create materials that can be utilized to replicate, enhance, repair, or replace human tissues and organs.^[3]^ Sports medicine addresses physical health issues, such as the management and avoidance of injuries brought on by activity and age-related conditions that impair the locomotor system.^[4–7]^ Thus, the structural and functional healing of various interconnected tissues and structures in the locomotor system is essential for the prevention and management of concerns in the clinical practice of sports medicine. When healing fails to restore structural integrity, strategies that promote tissue regeneration are needed to prevent or delay the use of non-organic structures, such as artificial joints. In order to restore the structural and functional integrity of the locomotor system, we then go on to the field of regenerative sports medicine, which is defined as a discipline of medicine that employs techniques to promote the regeneration of organs or tissue structures. Regenerative sports medicine today employs a variety of structurally leveled organic and non-organic material techniques. From a clinical perspective, regenerative sports medicine addresses problems related to aging and sports in various parts of the locomotor system, such as the menisci, ligaments, tendons, cartilages, and bones.

We analyzed the top 10 cited articles and found that regenerative medicine is mainly used for knee arthritis, which also proves from the side that knee arthritis is a common disease in athletes. Research in regenerative sports medicine is moving so fast that it is difficult to fully comprehend its state and key areas at this time. Bibliometrics is the quantitative analysis of certain subjects using a variety of databases, including Pubmed and Web of Science (WOS), among others.^[8,9]^ Among these, the Citespace software enables WOS to carry out quantitative scientific analysis.^[10,11]^ By using bibliometrics, readers may fully comprehend the frontiers, trends, and hotspots in this field.^[8,10]^ It looks at the turning points in the evolution of several topic areas. Bibliometrics includes coauthor, co-citation, and co-occurrence analysis.^[12,13]^

Because the subject of regenerative medicine is very complex and constantly evolving, often even contradicting itself, study findings are widely published as scholarly articles.^[14]^ On the one hand, cooperation between sport and regenerative medicine has advanced much faster thanks to the rise in published research. The vast amount of dispersed, perhaps redundant papers that comprise this topic makes it challenging to have a complete understanding of the state of research on regenerative medicine and sport today. There is not yet a bibliometric method for researching how sport and regenerative medicine interact. To this end, Citespace was used to investigate the global importance and trends of sport and regenerative medicine in the WOS database from January 1, 2013, to December 30, 2023.

## 2. Methods

### 2.1. Inclusion criteria

The inclusion criteria were as follows: basic and clinical research on regenerative medicine and sport; reviews on abdominal adhesions; and articles retrieved from the WOS.

### 2.2. Exclusion criteria

The exclusion criteria were as follows: articles that were not officially published; proceedings, conference abstracts, and corrigendum documents; unrelated articles; and duplicate articles.

### 2.3. Quality assessment

English-language articles that met the inclusion criteria were included in the analysis.

### 2.4. Source of literature

We enter the subject terms into the WOS database (SCI, SSCI, ESCI, etc): TS = ([Regenerative Medicine] OR [stem cells] OR [Medicine, Regenerative] OR [Medicine, Regenerative] OR [platelet-rich plasma]) AND TS = ([sporting] OR [sports] OR [sports]). The search scope of the database is from January 1, 2013, to December 30, 2023, and the language type was English. Through the literature search (articles, reviews, meeting abstracts, etc), 572 records were obtained. Shanxi Bethune Hospital database is the source of the WOS database. Every article that had the “search terms” has been examined (because not every article that was retrieved was 100% relevant). Separately, the 2 researchers screened and evaluated the literature. Bibliometric data, including publication date, number of publications/citations annually, countries/regions, institutions, authors, journals, references, keywords, and burst words, were provided by these publications.

### 2.5. Analysis software

Citespace analysis software version is Version 5.6. R2.^[12]^

### 2.6. Download and import of data

The file type remained “plain text” after the results of the retrieved topic terms were exported.^[11,15]^

### 2.7. Parameter setting

Fifty selection criteria, time slicing (from 2013 to 2023), individual node type checking, pathfinder pruning, and visualization (displaying the combined network, cluster view-static).

### 2.8. Statistical methods

**Figure 1.**
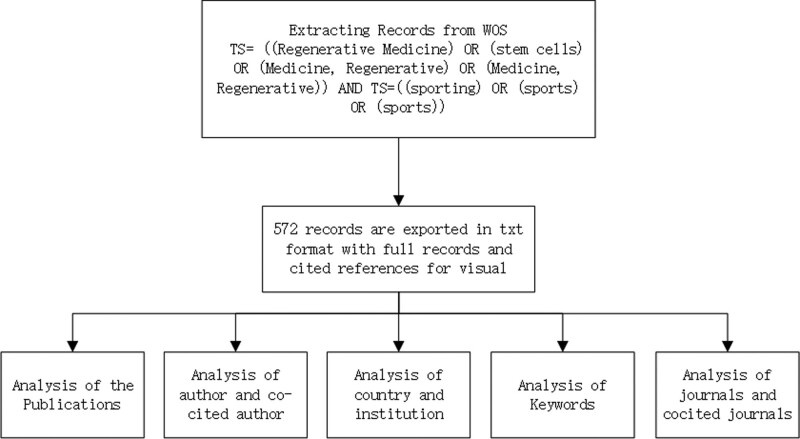
Analysis flowchart of sport and regenerative medicine. WOS = Web of Science.

Data management and annual publishing analysis were conducted using Microsoft Office Excel 2021. Every work of literature has undergone a scientific analysis; among the information gathered are key nations, organizations, writers, co-cited references, and co-occurrences of certain keywords.^[13,15,16]^ The detailed analysis flow is shown in Figure 1.

## 3. Results

### 3.1. Analysis of the publications

**Figure 2.**
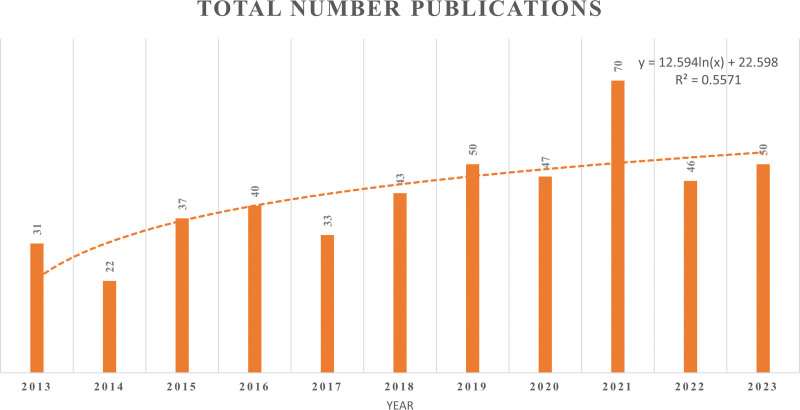
The number of sport and regenerative medicine publications indexed by WOS from 2013 to 2023. WOS = Web of Science.

The overall number of papers fluctuated during the research period. The research is broken down into 2 stages, as shown in Figure 2: the first stage spans the years 2013 to 2017 and the second stage spans the years 2018 to 2023. There was a surge in development during the second stage. In 2018, the magazine published 43 references; by 2021, that number had increased to 70. These results imply that the field of sport and regenerative medicine research has become more significant during the past 5 years.

### 3.2. Analysis of countries and institutions

**Figure 3.**
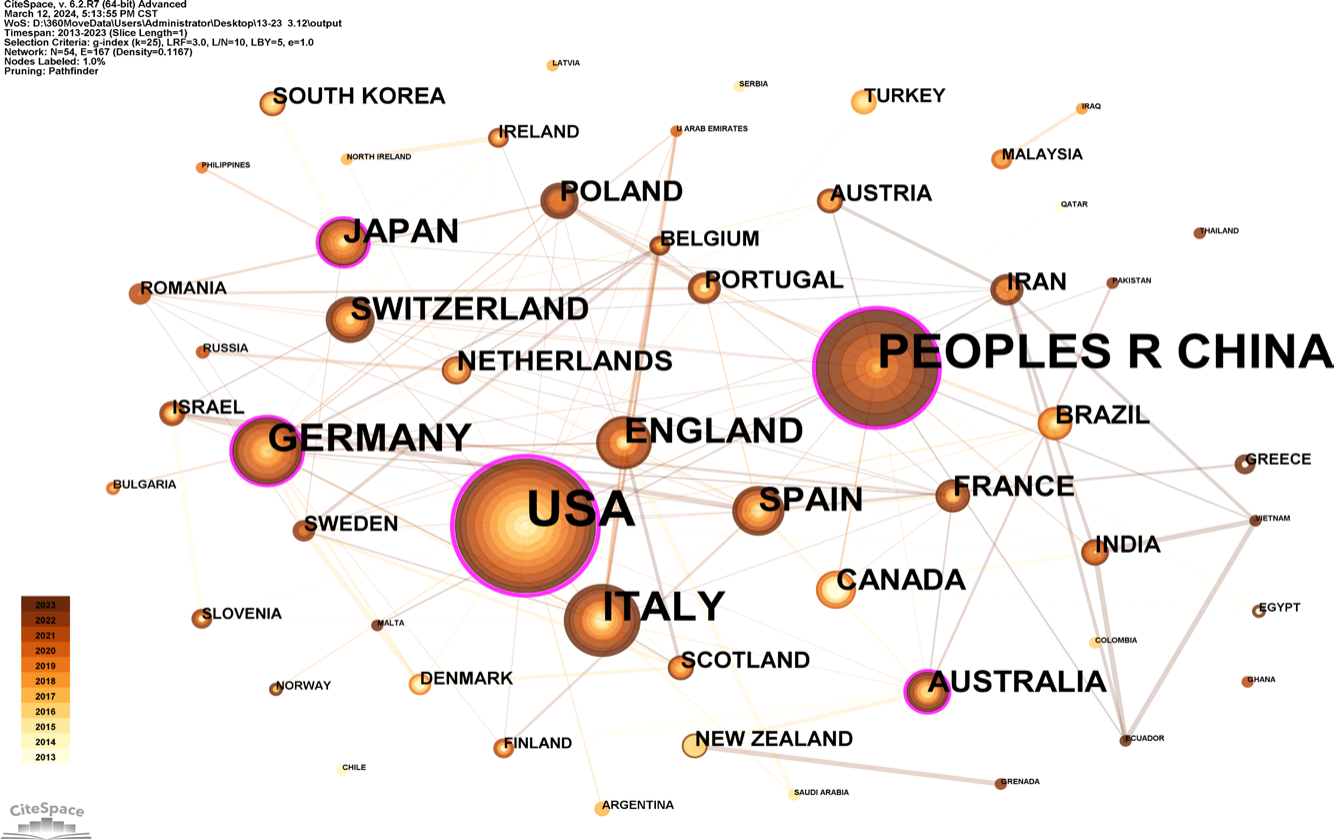
Analysis of the country map from 2013 to 2023.

A country map was generated (Fig. 3). Fifty-four countries published 658 references. The USA, the People’s Republic of China, Italy, Germany, and England are the top 5 countries (Table 1). The USA (0.53) and the People’s Republic of China (0.23) are the top 2 countries from centrality (purple round). The USA, the People’s Republic of China, and Italy were the primary research nations in the field of sport and regenerative medicine, according to a review of publications and centrality. The United States, the People’s Republic of China, and Germany have shown growing interest in this sector.

**Table 1:** Top 10 countries and institutions researching sport and regenerative medicine.

Ranking	Country	Publications	Ranking	Institution	Publications
1	USA	149	1	Harvard University	18
2	People’s Republic of China	110	2	University of London	17
3	Italy	55	3	Pennsylvania Commonwealth System of Higher Education (PCSHE)	14
4	Germany	40	4	Queen Mary University London	13
5	England	28	5	University of Pittsburgh	13
6	Spain	25	6	Steadman Philippon Research Institute	12
7	Japan	23	7	University of Salerno	11
8	Switzerland	20	8	University of California System	10
9	Australia	16	9	Peking University	10
10	Canada	15	10	Rutgers University System	8

**Figure 4.**
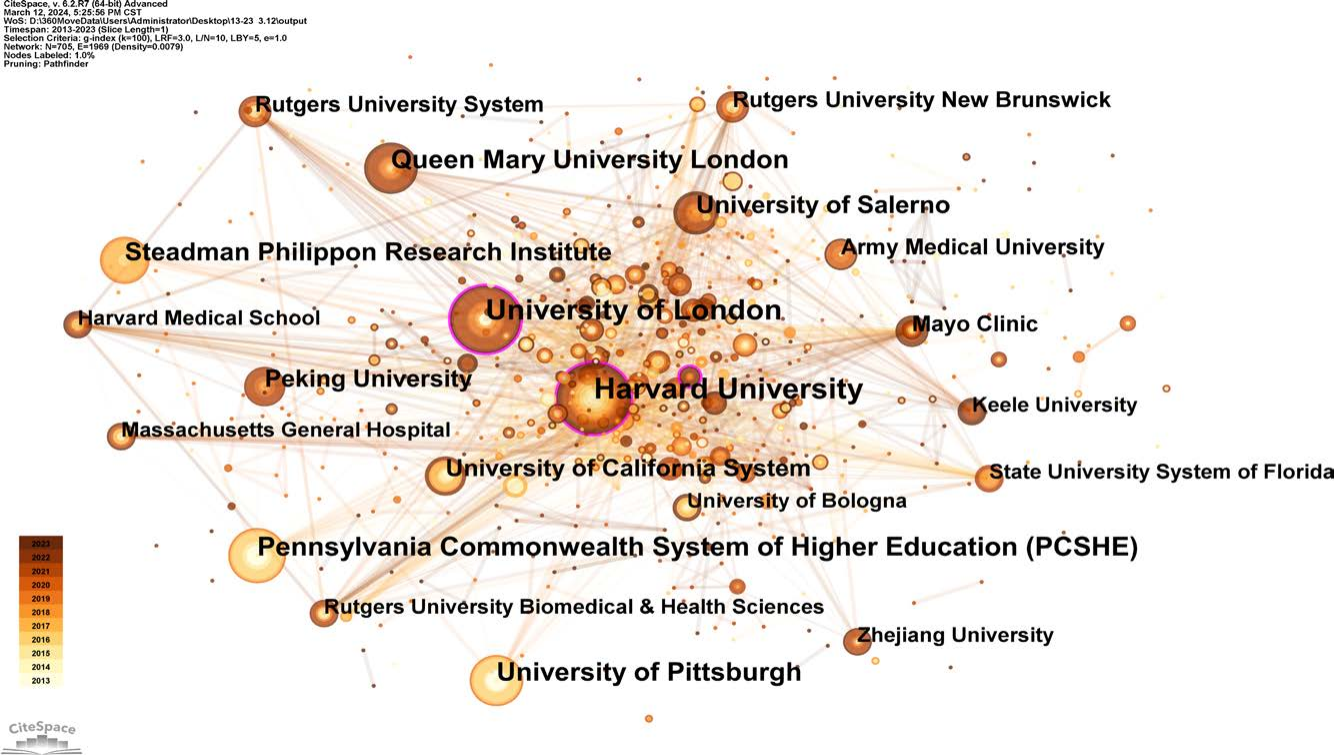
Institutional map researching sport and regenerative medicine from 2013 to 2023.

Generated an institution map with 705 nodes and 1969 links (Fig. 4). The 1273 publications have been published in 705 institutions. The Harvard University, the University of London, the Pennsylvania Commonwealth System of Higher Education, Queen Mary University London, and the University of Pittsburgh are the top 5 institutions (Table 1). Regarding centrality, the top 3 institutions were Harvard University (0.15), IRCCS Istituto Ortopedico Galeazzi (0.15), and the University of London (0.1).

Furthermore, we discovered that Peking University joined this sector in 2018 and has produced 10 articles in 5 years, demonstrating its ability to make contributions. Moreover, the relatively small number of linkages between various institutions suggests a lack of collaboration.

### 3.3. Analysis of the author

**Figure 5.**
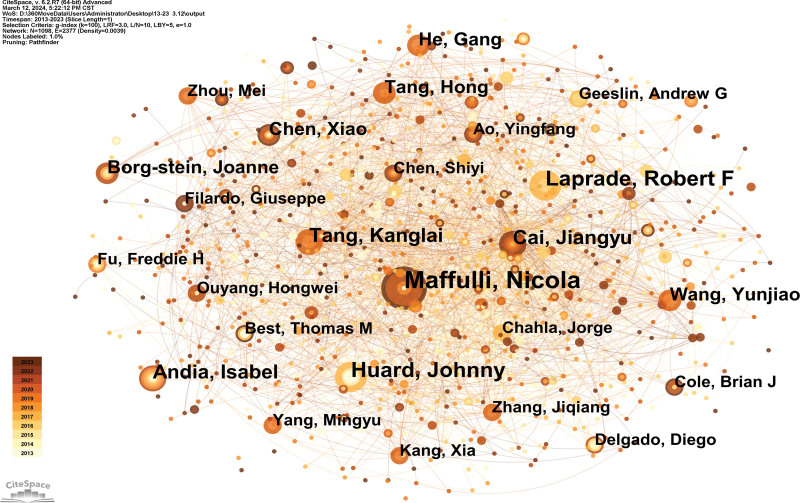
Author map researching sport and regenerative medicine from 2013 to 2023.

One thousand and ninety-eight authors published 1345 articles. The top 5 authors who have been written about (Table 2) are experts in this field. The generated map had 1098 nodes and 2377 links (Fig. 5).

**Table 2:** Top 10 authors in sport and regenerative medicine in terms of centrality.

Ranking	Cited reference	Centrality	Representative author (publication year)
1	10	0	Maffulli, Nicola (2013)
2	7	0	Huard, Johnny (2013)
3	7	3.52	Laprade, Robert (2016)
4	6	0	Cai, Jiangyu (2017)
5	6	3.06	Tang, Kanglai (2019)
6	6	0	Andia, Isabel (2013)
7	5	2.55	Wang, Yunjiao (2019)
8	5	2.55	He, Gang (2019)
9	5	2.55	Tang, Hong (2019)
10	5	0	Chen, Xiao (2014)

As the writer whose works have been published the most, Maffulli Nicola is based at the First Institute of Radiology in Italy. Their team could provide clinical support to the player, including regenerative medicine and stem cell^[16]^ (Table 2).

### 3.4. Analysis of co-cited references

**Figure 6.**
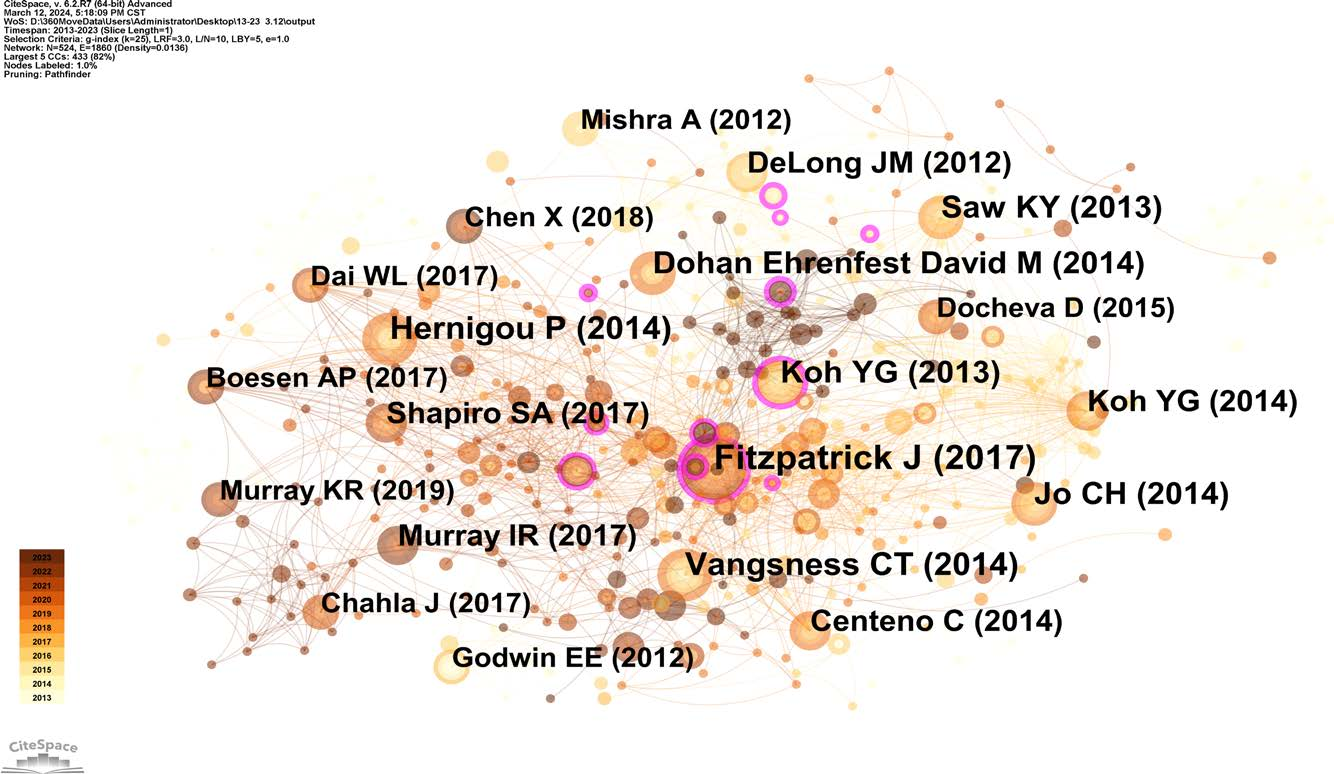
Co-cited references map researching sport and regenerative medicine from 2013 to 2023.

An analysis of counts and centrality (Fig. 6, Table 3) revealed that the data usually comes in the form of a review. Among them, “The Effectiveness of Platelet-Rich Plasma in the Treatment of Tendinopathy: A Meta-analysis of Randomized Controlled Clinical Trials” was published in *The American Journal of Sports Medicine* in 2016. Fitzpatrick J, FACSP, confirmed that there is good evidence to support the use of a single injection of leukocyte-rich PRP under ultrasound guidance in tendinopathy. Both the preparation and intratendinous injection technique of PRP appear to be of great clinical significance. It can be seen that, as the main classification of regenerative medicine, PRP is getting more and more attention in sports medicine. The second-ranked reference was released in 2014, and it showed that knee discomfort might be reduced and meniscus regeneration could occur after allogeneic human mesenchymal stem cell therapy. These findings bolster the investigation of human mesenchymal stem cells for their potential to protect and regenerate knee tissue.^[17]^

**Table 3:** Top 10 co-cited references related to sport and regenerative medicine research in terms of co-citation.

Ranking	Cited reference	Co-citation counts	Representative author (publication year)
1	The effectiveness of platelet-rich plasma in the treatment of tendinopathy: a meta-analysis of randomized controlled clinical trials	14	Fitzpatrick (2017)
2	Adult human mesenchymal stem cells delivered via intra-articular injection to the knee following partial medial meniscectomy: a randomized, double-blind, controlled study	12	Vangsness (2014)
3	Biologic augmentation of rotator cuff repair with mesenchymal stem cells during arthroscopy improves healing and prevents further tears: a case-controlled study	12	Hernigou (2014)
4	Mesenchymal stem cell injections improve symptoms of knee osteoarthritis	10	Koh (2013)
5	Intra-articular injection of mesenchymal stem cells for the treatment of osteoarthritis of the knee: a proof-of-concept clinical trial	10	Jo (2014)
6	Articular cartilage regeneration with autologous peripheral blood stem cells versus hyaluronic acid: a randomized controlled trial	10	Saw (2013)
7	Efficacy of autologous bone marrow concentrate for knee osteoarthritis with and without adipose graft	9	Centeno (2014)
8	A prospective, single-blind, placebo-controlled trial of bone marrow aspirate concentrate for knee osteoarthritis	9	Shapiro (2017)
9	Minimum information for studies evaluating biologics in orthopaedics (MIBO): platelet-rich plasma and mesenchymal stem cells	9	Murray (2017)
10	Platelet-rich plasma: the PAW classification system	9	DeLong (2012)

### 3.5. Analysis of keyword co-occurrence and burst analysis

**Figure 7.**
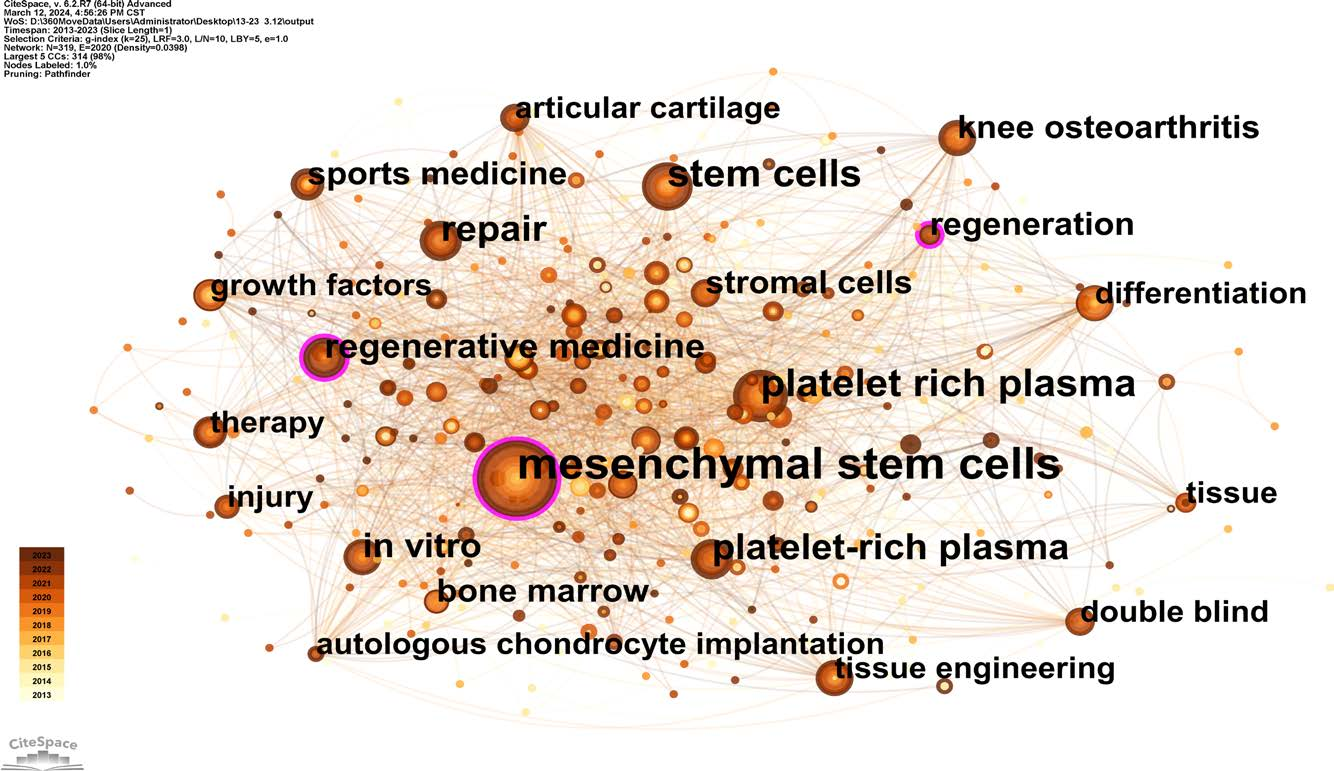
Co-occurrence keyword map researching sport and regenerative medicine from 2013 to 2023.

A co-occurrence map of keywords could show popular subjects. A term co-occurrence map that was created contained 2020 linkages and 319 nodes (Fig. 7). It demonstrates that mesenchymal stem cells, platelet-rich plasma, stem cells, repair, and platelet-rich plasma were the most often used keywords.

**Figure 8.**
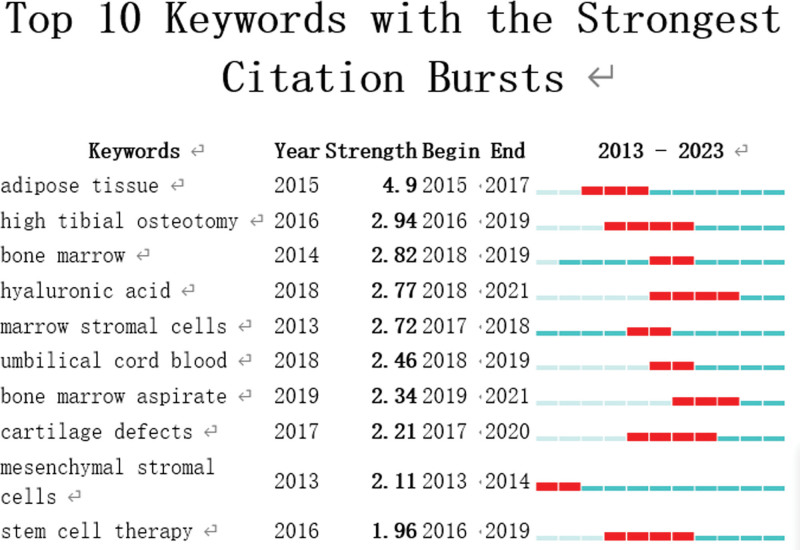
Top 10 keywords with the strongest citation bursts.

“Burst words” are words that are frequently used over long periods of time. It may be possible to predict the frontier of research trend by looking at the distribution of terms with the strongest citation explosion. The top 10 terms with the biggest increase in citations between 2013 and 2023 are shown in Figure 8. The keyword was cited occasionally, as indicated by the green bars, and frequently, as represented by the red bars. It is possible to come across references to adipose tissue, high tibial osteotomy, bone marrow, hyaluronic acid (HA), marrow stromal cells, umbilical cord blood, and bone marrow aspirate, which suggests that interest in this field of study will only increase in the years to come.

## 4. Discussion

Citespace data indicates that between 2018 and 2023, there were more annual articles about sports and regenerative medicine. That may be because regenerative medicine—a vital component of sports—is receiving more attention from researchers. Probably because of the publication of “The Effectiveness of Platelet-Rich Plasma in the Treatment of Tendinopathy: A Meta-analysis of Randomized Controlled Clinical Trials,”^[18]^ it has been cited 98 times. This article helps supporters and members of the American Orthopaedic Society for Sports Medicine stay informed on the most recent research in the field of sports regenerative medicine. The greater quantity of articles on sports regenerative medicine that we identified in the United States may be attributable to Chinese sports research’s difficulty in producing English-language articles. China stands second, surpassed only by the United States. Notably, it is the only developing nation within this ranking. This may be attributed to the larger number of research in China.

We can see the importance of stem cells in regenerative sports medicine, but before stem cell therapies can live up to the high expectations placed on them, much more research is needed. The International Society for Stem Cell Research (ISSCR) provides a clear explanation of the risks and unproven efficacy of their treatments, so interested parties can avoid spending money on unproven, expensive cell therapies (ISSCR, 2013).^[19]^ Also, a set of performance guidelines is available for responsible administration of stem cell therapies (ISSCR, 2008).^[20]^ It is now widely acknowledged that physical activity offers a pertinent stimulus to bring about transformation, which in turn primes or activates adult stem cells.^[21]^ Barrett et al^[22]^ initially reported on the possible beneficial effects of exercise on the mobilization of hematopoietic stem cells in the late 1970s. Further research revealed that the process depended on time, intensity, and age. About 3 decades later, in the late 2000s, a plethora of research on the topic of exercise and stem cells emerged, covering all the systems that are involved in exercise adaptation.

The Harvard University is the most influential in the field of regenerative medicine, it includes some highly cited articles, that is, “Mesenchymal stem cells in regenerative medicine: Focus on articular cartilage and intervertebral disc regeneration” has been cited 202 times. These highly cited articles not only make Harvard University the most published institution, but also have a very large influence in the field.

Regenerative sports medicine is essential for treating disorders and injuries involving the locomotor system since sports medicine has a high requirement for tissue regeneration procedures. However, there are a few issues that are restricting the use of these methods. The first challenge is like-for-like restoration, or the creation of tissues that, in terms of both form and function, resemble the original tissues. The second problem is how to create a structure that can make up for the original structure’s shortcomings so that both its functionality and the risk of reinjury are increased. Surgeons around the world are facing more and more clinical issues as sports medicine injuries become more common. The field of regenerative sports medicine will offer encouraging possibilities for the regeneration of the locomotor system’s structure and functionality. In contrast to tissue regeneration in other bodily systems, the structure to be regenerated in the locomotor system should be able to withstand a variety of forces during regeneration without clearly deforming and should reestablish the original connection between the regenerated structure and the various surrounding tissues. Sports medicine injury repair scaffolds of the future depend on the use of the best structural material components, bionic 3D architectures that mimic natural structures, and the long-term viability of the bio-actively regenerated structure.

Adipose tissue, high tibial osteotomy, bone marrow, HA, marrow stromal cells, differentiation, umbilical cord blood, and bone marrow aspirate may be quoted frequently, according to “Burst words,” suggesting that interest in this field of study will likely grow in the years to come. PRP (and its derivatives) and HA have been thoroughly studied in the literature for the treatment of musculoskeletal conditions of the knee, particularly in terms of degenerative joint.^[23,24]^ Several studies^[18,25,26]^ have shown positive results when using both PRP and HA; however, PRP may or may not occasionally show to be marginally better than HA. PRP can have a variety of effects, such as neovascularization, cytokine release, differentiation, recruitment of new cells, and control of inflammation.^[27]^ Likewise, HA reduces inflammation, promotes anabolic responses, increases lubrication, enhances biomechanics, and promotes cell migration, proliferation, and differentiation.^[28]^ When taken as a whole, these reactions help to heal injured musculoskeletal tissues, such as cartilage, tendons, and bone, and to reduce pain.

## 5. Limitations

Analytical and visualization tools, such as CiteSpace, offer an expansive perspective on the progression and trajectory of vasculitis immunotherapy. Nevertheless, this study has some inherent limitations. The range of literature considered is not exhaustive since it only integrates data from the WOS, omitting information from other relevant databases. Moreover, the selection of literature is limited solely to English publications, thereby inducing language bias. Thus, the analyzed literature may not thoroughly reflect all studies pertaining to vasculitis immunotherapy.

## 6. Conclusion

The USA, the People’s Republic of China, and France have emerged as the 3 main research nations in this field thanks to their high publication rates and centrality. Research on sports and regenerative medicine benefits from the best partnerships between developed countries and esteemed universities. Because these articles have a high impact factor or serve as guidelines, they were frequently mentioned.

## Acknowledgments

The authors would like to express their appreciation to Professor Chaomei Chen for inventing Citespace and making it free to use.

## Author contributions

**Conceptualization:** Lv Xiongce, Lixia Wang.

**Project administration:** Lv Xiongce.

**Writing – original draft:** Lv Xiongce, Yan Jin, Lixia Wang.

**Formal analysis:** Ye Tao, Yan Jin.

**Investigation:** Ye Tao.

**Resources:** Jing Zhu.

**Software:** Jing Zhu.

**Data curation:** Lixia Wang.

**Writing – review & editing:** Lixia Wang.
